# Supplementary material for: Construction of Tissue-Engineered Bladder Scaffolds with Composite Biomaterials
Source: Polymers (Basel). 2022 Jun 29;14(13):2654. doi: 10.3390/polym14132654 (PMC9269300; doi:10.3390/polym14132654)
Supplement: Supplementary file 1 [file polymers-14-02654-s001.zip › polymers-1753690-SI.pdf]

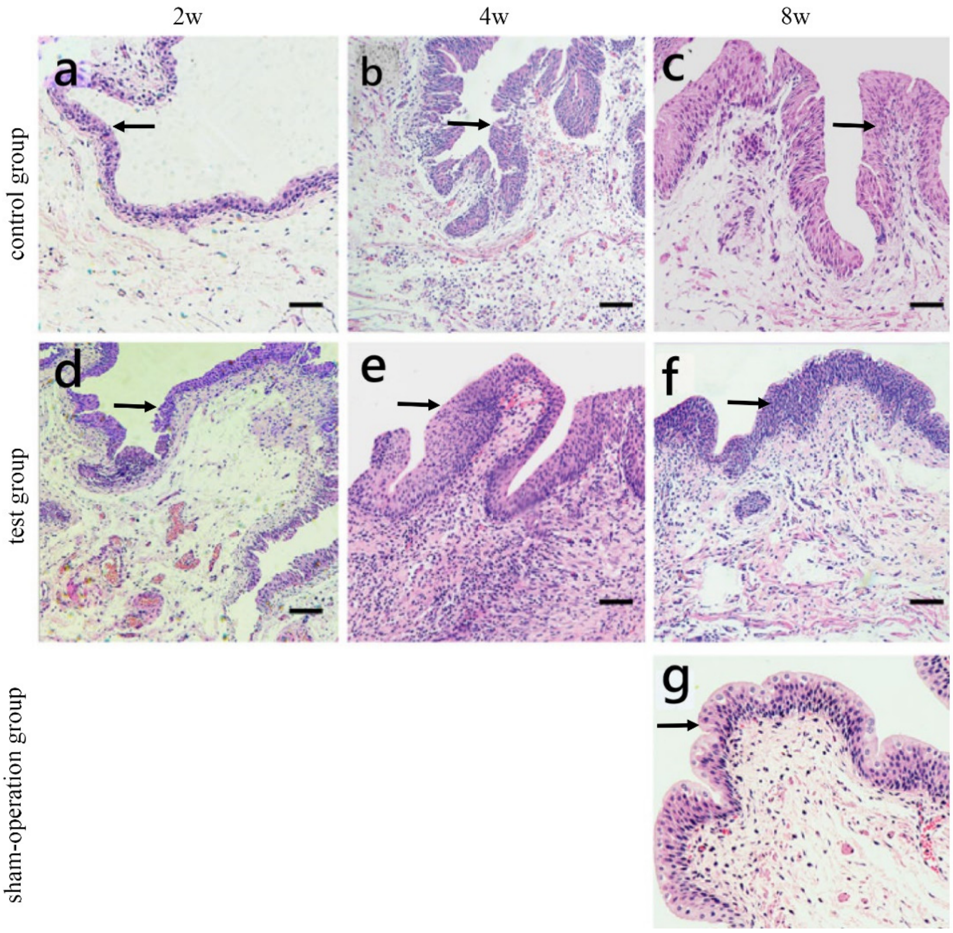

Figure S1. The cell growth by haematoxylin and eosin (H&E) staining. H&E staining showed that with the passage of time (2w ~ 8w), the number of urothelial cells migrating to the marginal zone and those migrating to the central zone gradually increased.
